# Human Influenza A (H5N1) Cases, Urban Areas of People’s Republic of China, 2005–2006

**DOI:** 10.3201/eid1307.061557

**Published:** 2007-07

**Authors:** Hongjie Yu, Zijian Feng, Xianfeng Zhang, Nijuan Xiang, Yang Huai, Lei Zhou, Zhongjie Li, Cuiling Xu, Huiming Luo, Jianfeng He, Xuhua Guan, Zhengan Yuan, Yanting Li, Longshan Xu, Rongtao Hong, Xuecheng Liu, Xingyu Zhou, Wenwu Yin, Shunxiang Zhang, Yuelong Shu, Maowu Wang, Yu Wang, Chin-Kei Lee, Timothy M. Uyeki, Weizhong Yang, for the Avian Influenza H5N1 Study Group

**Affiliations:** * Chinese Center for Disease Control and Prevention, Beijing, People’s Republic of China; † Hubei Center for Disease Control and Prevention, Wuhan, People’s Republic of China; ‡ International Field Epidemiology Training Program, Bangkok, Thailand; § National Institute for Viral Disease Control and Prevention, Beijing, People’s Republic of China; ¶ Guangdong Center for Disease Control and Prevention, Guangzhou, People’s Republic of China; # Shanghai Center for Disease Control and Prevention, Shanghai, People’s Republic of China; ** Fujian Center for Disease Control and Prevention, Fuzhou, People’s Republic of China; †† Sichuan Center for Disease Control and Prevention, Chengdu, People’s Republic of China; ‡‡ Shenzhen Center for Disease Control and Prevention, Shenzhen, People’s Republic of China; §§ World Health Organization, Beijing, People’s Republic of China; ¶¶ Centers for Disease Control and Prevention, Atlanta, Georgia, USA

**Keywords:** Avian influenza, H5N1, wet poultry market, dispatch, China

## Abstract

We investigated potential sources of infection for 6 confirmed influenza A (H5N1) patients who resided in urban areas of People’s Republic of China. None had known exposure to sick poultry or poultry that died from illness, but all had visited wet poultry markets before illness.

Although >280 confirmed human cases of avian influenza A (H5N1) virus infection from 12 countries have been reported (*1*), detailed data on sources of infection for most patients are limited (*2*). In Vietnam, 8 of 9 patients with influenza A (H5N1) reported close contact with sick or dead poultry (*3*). In Thailand, 9 of 12 such patients lived in households where backyard chickens died, and 8 reported direct contact with dead chickens (*4*). Case-control studies in Thailand and Vietnam found that the most statistically significant risk factor was recent exposure to sick or dead poultry, especially directly touching dead poultry (*5*,*6*).

Avian influenza (H5N1) poultry outbreaks have been reported in mainland People’s Republic of China since 2004 (*7*); since late 2005, human cases have also been reported (*8*). Most Chinese patients had exposure to backyard poultry, although some had no apparent direct exposure to poultry that were sick or died. We describe findings of investigations of urban patients with influenza A (H5N1), who had no known direct contact with sick poultry or poultry that died of illness in China.

## The Study

Enhanced surveillance for influenza-like illness and pneumonia of unknown origin was established in China after the outbreak of severe acute respiratory syndrome (SARS) (*9*). All suspected cases of influenza A (H5N1) are reported through a national surveillance system to the Chinese Center for Disease Control and Prevention (China CDC). Laboratory testing is performed by the National Influenza Center of China CDC. A confirmed case of influenza (H5N1) was defined according to World Health Organization case definitions (*10*). This study was part of an ongoing public health outbreak investigation and determined by the ministry of health to be exempt from institutional review board assessment.

China CDC conducted epidemiologic investigations by interviewing confirmed influenza (H5N1) patients and their relatives, reviewing medical records, and visiting patient households and places visited by patients within 2 weeks of illness onset. A standardized questionnaire was used to collect demographic, clinical, and exposure history data. For 3 severely ill patients, only relatives and contacts were interviewed to assess possible influenza (H5N1) subtype exposures. Particular attention was paid to potential exposures such as contact with well-appearing, sick, or dead poultry; visits to poultry markets; or contact with persons with febrile respiratory symptoms in the 2 weeks before onset. A rural case was 1 that occurred in a village resident; an urban case was 1 that occurred in a city resident.

**Figure 1:**
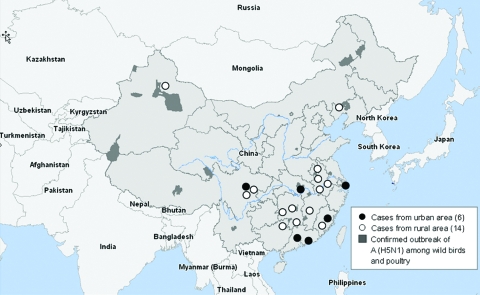
Map showing distribution of 6 human influenza (H5N1) cases from urban areas of People’s Republic of China, compared with 14 cases from rural areas. The 6 urban cases were distributed sporadically in 6 large cities of 5 provinces, and none was associated with confirmed H5N1 subtype poultry outbreaks or sick and dead poultry.

**Figure 2:**
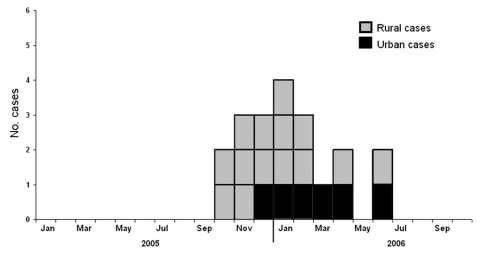
Number of human influenza (H5N1) cases in urban and rural areas, by month of onset, People’s Republic of China, October 2005–September 2006.

From October 2005 through October 2006, 20 confirmed cases were reported from 12 provinces. Six cases were identified in 6 cities of 5 provinces; each city had an average population of 8.3 million and was at least 112 km away from cities with another case (Figure 1). More urban cases were reported in 2006 (5 [42%] of 12) than in 2005 (1 [13%] of 8), but this difference was not statistically significant (p = 0.325) (Figure 2). Demographic and clinical characteristics of the 6 urban patients with influenza (H5N1) cases are presented in Table 1. All 6 urban patients were adults, median age 30 years (range 21 to 41); 5 died.

**Table 1:** Characteristics of 6 human influenza A (H5N1) case-patients from urban areas of China

Characteristic	Case-patient					
	1	2	3	4	5	6
Location (city, province)	Sanming, Fujian	Chengdu, Sichuan	Guangzhou, Guangdong	Shanghai, Shanghai	Wuhan, Hubei	Shenzhen, Guangdong
Residence	Apartment	Apartment	Apartment	Temporary, at construction site	Dormitory	Apartment
Illness onset date	Dec 6, 2005	Jan 10, 2006	Feb 22, 2006	Mar 13, 2006	Apr 1, 2006	Jun 3, 2006
Days from onset to investigation	6	7	6	8	10	8
Contact with ill persons before onset	No	No	No	No	No	No
Days from onset to first medical visit	2	4	3	2	5	4
Days from onset to hospital admission	3	7	5	9	11	7
Primary signs and symptoms at admission	Fever, headache, cough	Fever, cough, diarrhea, myalgia	Fever, chills, cough, shortness of breath	Fever, chills, cough	Fever, headache, sore throat, cough, myalgia	Fever, chills, productive cough, back pain
Complications*	Respiratory failure, ARDS, cardiac failure, septicemia	Respiratory failure, ARDS, cardiac failure, septicemia	Respiratory failure, ARDS	Respiratory failure, ARDS, cardiac failure	Respiratory failure, ARDS, cardiac failure, septicemia	Respiratory failure, ARDS
Outcome	Died	Died	Died	Died	Died	Survived
Days from onset to death	16	14	9	9	19	Discharged 61 d from onset

*Respiratory failure was defined as the need for ventilator support; ARDS, acute respiratory distress syndrome, is a life-threatening condition in which inflammation of the lungs and accumulation of fluid in the air sacs (alveoli) lead to low blood oxygen levels; cardiac failure was defined as the requirement of isotropic agents.

Five of the 6 urban case-patients had no direct contact with poultry. One patient prepared freshly slaughtered chicken that she purchased for cooking at a live (wet) poultry market. No patients kept poultry or other animals at home, and no poultry or poultry outbreaks were identified in their neighborhoods. Five patients had visited wet poultry markets within a week of illness onset, and all had visited a wet market during the 2 weeks before their illness. Three patients visited wet markets at least once a day before illness onset. Only 1 patient (case-patient 5) had any travel history in the 2 weeks before illness onset. That patient had visited his parents’ home in a rural area, where healthy backyard poultry were kept outside the house, and he had visited a wet market in the same area 2 weeks before illness onset (Table 2).

**Table 2:** Exposure history of 6 influenza A (H5N1) case-patients from urban areas of China

No.	Epidemiologic information
1	41-y-old female factory worker, previously healthy, with a history of thymectomy for benign thymoma 6 wk before illness onset, had recovered fully, did not require any medications, and resumed working. She visited a wet market nearly every day the week before illness onset but did not purchase poultry or poultry products. No poultry were kept in her home or neighborhood.
2	29-y-old woman, previously healthy, worked at a stall that she owned at a wet market, selling groceries and eggs. Her stall was ≈20 m away from stalls selling and slaughtering live poultry. No poultry were kept in her home or neighborhood.
3	32-y-old man, previously healthy, quit his job 1 mo before illness onset and was planning to start his own food business. He visited up to 9 wet markets for 10–40 min every day during the week before illness onset. At 1 wet market, he spent most of his time in a sauce store that was ≈5 m away from stalls where poultry were slaughtered and sold. No poultry were kept in his home or neighborhood.
4	29-y-old woman, previously healthy, moved from Guangdong to Shanghai 2 mo before illness onset. She worked as a cook for 14 people at a construction site, where she lived temporarily, and visited a wet market every day to buy fresh food, including freshly slaughtered chickens, 1 wk before illness onset. No poultry were kept in her home or neighborhood.
5	21-y-old man, previously healthy, was a security guard for an aircraft-repairing factory in Wuhan. Two wk before illness onset, he traveled to his hometown in the rural area of Enshi to attend the funeral of his uncle, who died of esophageal cancer. The man stayed there for 6 d, visited his parents’ home, where healthy backyard poultry were kept (none became sick or died), and visited a wet market. One wk before onset, he traveled back to his workplace in Wuhan, bringing 200 eggs from his hometown. In Wuhan, he had no direct contact with poultry, and he did not visit any wet markets. No poultry were kept in his home or neighborhood.
6	31 y-old man, previously healthy, worked as a truck driver for shoe factories in Shenzhen city. Two d before illness onset, he visited a wet market once, but he did not purchase any poultry or poultry products. One wk before onset, his wife visited the same market and brought a live chicken that was slaughtered at the market. No abnormal dieoffs of poultry were reported. No poultry were kept in his home or neighborhood.

All 6 patients had no known contact with other confirmed influenza A (H5N1) patients or with anyone with febrile respiratory symptoms. A total of 640 persons were followed up for medical observation for 2 weeks, including 136 close contacts of the 6 patients, 389 healthcare workers who provided care for them, and 115 persons who worked in the poultry markets visited by the patients. Febrile respiratory illness developed in 5 contacts: case-patient 1’s mother, case-patient 3’s girlfriend, case-patient 6’s daughter, a nurse who cared for case-patient 6, and a patient hospitalized on the same ward as case-patient 6. All 5 ill contacts recovered, and all respiratory specimens collected from them tested negative for influenza A (H5N1) by reverse-transcriptase–PCR. Paired acute- and convalescent-phase serum samples collected from these 5 ill contacts tested negative for subtype (H5N1) neutralizing antibodies by microneutralization assay.

## Conclusions

Our study suggests that exposure to wet poultry markets may be an important influenza A (H5N1) risk factor for persons in urban areas of China. None of the 6 case-patients had known direct contact with poultry that were sick or died of illness. Two patients (case-patients 1 and 3) had no identified potential exposures except for visiting a wet poultry market during the week before illness onset. Four other case-patients visited wet markets, although other exposures could have potentially led to virus transmission. Case-patient 2 was an egg seller and could have also been infected by contact with fecally contaminated eggs. In 2005, influenza A (H5N1) virus was isolated from eggs brought to China by travelers from Vietnam (*11*). Case-patient 4 could potentially have been exposed to the virus through preparation of freshly slaughtered chickens purchased at a wet market. Case-patient 5 could have been exposed to the virus by visiting his parents’ home, which had healthy backyard poultry outside, or by transporting eggs. Case-patient 6 could have been exposed to the virus at home when his wife prepared a freshly slaughtered chicken purchased from a wet market. No epidemiologic evidence suggested human-to-human transmission of influenza A (H5N1) associated with the urban patients.

These observations are consistent with results of a case-control study conducted during the 1997 influenza A (H5N1) outbreak in Hong Kong Special Administrative Region, which found the most statistically significant influenza (H5N1) risk factor was visiting a live poultry market the week before illness onset (*12*). During that outbreak, widespread subtype (H5N1) poultry dieoffs occurred in wet markets, but these dieoffs have not been observed in urban China. The role of poultry vaccination in decreasing poultry outbreaks in wet markets in China is unknown. A recent simulation study showed that silent spread of the virus can occur in poultry because of incomplete protection at the flock level, even if a poultry vaccine is effective in individual birds (*13*).

In China, wet markets are sustained by demand for freshly slaughtered poultry. Wet markets are considered a reservoir and amplifier of avian influenza A viruses because they bring together avian host species in a high-density setting that can facilitate viral persistence, cross-species infection, and genetic reassortment (*14*). Our findings suggest that wet markets pose a risk that is likely to be low for avian-to-human transmission of subtype (H5N1) in urban settings. Viral RNA for subtype (H5N1) was detected in a specimen collected from a goose cage at an urban wet market visited by case-patient 3 (*15*), which suggests the potential for influenza (H5N1) transmission through environmental contamination.

We were limited by inability to elicit complete exposure histories from all case-patients because of their severe illness or death. However, we interviewed household and family members, friends, and co-workers, and investigated places that patients had visited in the 2 weeks before illness onset. We were unable to ascertain how human infection with the virus occurred through the patients’ visits to wet poultry markets. Possibilities include self-inoculation of the respiratory tract after touching subtype (H5N1)–contaminated surfaces, or inhalation of aerosolized debris with influenza (H5N1) virus.

We did not perform testing on poultry or environmental specimens and can only speculate about potential exposures and sources of influenza A subtype (H5N1) infection in wet markets, homes, and neighborhoods. Future studies should test tracheal, cloacal, and blood specimens from poultry; swabs of fecal material, cages, and other potentially contaminated surfaces; and air specimens in wet markets, for evidence of influenza (H5N1). More research studies, including case-control studies, are needed to better clarify the risk for subtype (H5N1) transmission that occurs from visiting wet poultry markets. Close collaborations are needed between animal health and public health agencies to reduce the public health risk for this virus in wet poultry markets and to understand the impact of poultry vaccination on the risk for transmission.
